# Complete Genome Sequence of *Bifidobacterium longum* GT15: Unique Genes for Russian Strains

**DOI:** 10.1128/genomeA.01348-14

**Published:** 2014-12-18

**Authors:** Natalia V. Zakharevich, Olga V. Averina, Ksenia M. Klimina, Anna V. Kudryavtseva, Artem S. Kasianov, Vsevolod J. Makeev, Valery N. Danilenko

**Affiliations:** a Vavilov Institute of General Genetics, Moscow, Russia; b Engelhardt Institute of Molecular Biology, Moscow, Russia

## Abstract

In this study, we report the first completely annotated genome sequence of the Russian-origin *Bifidobacterium longum* subsp. *longum* strain GT15. We discovered 35 unique genes (UGs) which were detected from only the *B. longum* GT15 genome and were absent from other *B. longum* strain genomes (not of Russian origin).

## GENOME ANNOUNCEMENT

Bifidobacteria represent an important group of the human intestinal microbiota (1). There is an increasing interest in the positive effects of bifidobacteria on the central nervous system (CNS) via neural, neuroendocrine (2), neuroimmune, and humoral mechanisms (3–5). Here, we present the first complete annotated genome sequence for Russian-origin strain *Bifidobacterium longum* subsp. *longum* GT15. This strain was isolated from the feces of a healthy adult inhabiting Central Russia and demonstrated good probiotic properties.

The complete genome of *B. longum* GT15 is composed of a 60% G+C circular chromosome of 2,337,521 bp with no plasmids. A total of 1,893 coding sequences (CDS) were predicted in the genome. Based on a Clusters of Orthologous Groups (COG) functional classification (6, 7), the highest number of genes associated with metabolism in strain GT15 belonged to the carbohydrate metabolism [G] category, which amounted to more than 10%. The GT15 genome contains 5 rRNA operons, 56 tRNA genes, 26 pseudogenes, and 114 tandem repeats. Nine phage-related fragments were predicted in the genome of *B. longum* GT15, but no complete prophages were found. The GT15 chromosome also possesses 13 complete or disrupted insertion sequence (IS) elements (belonging to IS*21*, IS*256*, IS*3*, IS*30*, and IS*L3* families). One confirmed cluster of regularly interspaced short palindromic repeat (CRISPR) (24-bp CRISPR repeat) and three potential CRISPR-related systems were discovered in the GT15 genome. In our analysis, we also examined genes for global regulatory systems; we annotated six genes of toxin-antitoxin (TA) systems type II (8), six serine/threonine protein kinases (STPKs) of eukaryotic type (9), and three genes of the WhiB-like family (10, 11). In general, comparative analysis indicated that the *B. longum* GT15 genome was similar to the available genomes. However, in the *B. longum* GT15 genome, we detected unique nucleotide sequences (more than 150 nucleotides in length) present only in the GT15 genome, and not found in any of the sequenced members of the *B. longum* species (not of Russian origin). We identified open reading frames (ORFs) within such sequences and called them unique genes (UGs). The GT15 genome contained 35 ORFs of such genes, of a total length of 39,066 bp, with G+C composition in the range of 37% to 65%. It is worth noting that all unique genes, except one, have a G+C composition lower than that of the overall genome of strain GT15. The genes encoded proteins of lengths varying from 57 to 1,218 amino acid residues. The 21 genes were similar to genes of *B. longum* subsp. *longum* strains of Russian origin: 1-6B, 2-2B, 44B, and 35B (12). Some of the unique genes are adjacent to each other, forming islands (clusters). In the genome of GT15, we found seven such gene clusters consisting of unique genes. These clusters include from two to seven genes. In addition, some of the clusters are flanked by different mobile elements and display significant divergence from the average G+C genome content or atypical codon usage, suggesting acquisition through horizontal gene transfer (HGT). A large proportion of these identified UGs encode hypothetical proteins with unknown function; thus, they may represent novel biosynthetic or human gut commensal interaction features.

### Nucleotide sequence accession number.

This complete genome project has been deposited in GenBank under the accession no. CP006741 and submitted for automated annotation at the National Center for Biotechnology Information (NCBI). The genome annotation was conducted with the NCBI Prokaryotic Genomes Automatic Annotation Pipeline (PGAAP) (http://www.ncbi.nlm.nih.gov/genome/annotation_prok/).
